# Putting China on the couch: Reflections on the development of psychohistory in China

**DOI:** 10.3389/fpsyg.2022.1010110

**Published:** 2022-12-05

**Authors:** Fei Meng, Bo Wang, Jian Chen

**Affiliations:** ^1^School of Marxism, Central China Normal University, Wuhan, China; ^2^Department of Philosophy, Xiamen University, Xiamen, China

**Keywords:** psychohistory, psychohistory in China, indigenization, psychohistory of China, Chinese psychology, Chinese mentality

## Abstract

In the process of reconstructing the history of Chinese psychology, psychohistory once drew little attention. Although applying psychological tools to historical studies has not been a new research method for Chinese historians, when it comes to psychohistory in its modern sense, it inevitably sounds exotic and novel to Chinese academia. However, the significance of psychohistory, especially the one with practical relevance, should not be underestimated. Thus, the history and the deficiency of psychohistory need to be clarified. Based on the macro-historical logic, the development of psychohistory in China can be recounted and divided into four stages, namely (1) before 1902, the pre-scientific stage of psychohistory, (2) 1902–1949, the introduction of modern psychohistory, (3) 1949–1978, the tortuous and lopsided development of psychohistory, and (4) 1978–present, the revival of diverse approaches in psychohistory. The possibilities of psychohistory as we find in such a process, in all its reality, reside in the fact that it could combine the history of ideas with reality and the history of society with ideas, which would undoubtedly improve our understanding of the intertwinement of the human psyche and the social mechanisms, in brief, the historical dynamics. In addition, psychohistory could also help solve psychological problems that the populations in modern times are currently facing. Despite all of these virtues, in terms of indigenization (particularization), generalization (universalization), trans-regional communication, and disciplinary institutionalization, there is still some way for psychohistory in China to go.

## The development of western psychohistory in the eyes of Chinese researchers

Using psychological methods in historical studies is a trend that originated in the West in the 20th century, and today, doing empirical historical research guided and interpreted by psychological insights is more than a distinctive and widely accepted approach named psychohistory (Belzen, 2013). Noland's view (1977) still holds value today. He stressed that psychohistory is a fashionable term with a definite purpose, but it has different meanings and uses. Sigmund Freud, the founder of psychoanalysis, was regarded as the first person who applied psychological methods to the study of historical figures. His book *Leonardo da Vinci: A Psychosexual Study of an Infantile Reminiscence* was generally considered the prototype of psychohistory. In the United States, psychohistory aroused huge attention among historians. In 1913, Preserved Smith's article “Luther's Early Development in the Light of Psychoanalysis” was considered the first serious attempt by a professional American historian in the field of psychohistory (Zhou, 2001, p. 52). From the 1950 to 1970's, psychohistory in America developed rapidly and eventually matured as a discipline with its own boundaries. A huge number of books on psychohistory were published during this period of time. Most of them were in the form of psychobiography, group psychohistory, or history of childhood and family, while psychoanalysis was still the most frequently used methodology. Among these books, *Young Man Luther: A Study in Psychoanalysis and History* (Erikson, 1958/1993), *The New Psychohistory* (deMause, 1975/1989), *Rousseau and the Spirit of Revolt: A Psychological Study* (Blanchard, 1967), *The Mind of Adolf Hitler: The Secret Wartime Report* (Langer, 1972), and *Stalin as Revolutionary, 1879-1929: A Study in History and Personality* (Tucker, 1973) were the few works on psychohistory translated into Chinese and were thus available to Chinese readers. In the meanwhile, several journals regarding psychohistory were also established. For Chinese readers, *The Journal of Psychohistory*, founded by Lloyd deMause, is the most influential one. Other important journals such as *The Journal of Interdisciplinary History* and *The American Historical Review*, which sometimes publish articles concerning psychohistory, also draw much attention.

France is another country that Chinese researchers pay attention to in the field of psychohistory. With the rise of the Annales School and New Cultural History approach, psychohistory in France developed in the form of “histoire des mentalités” (history of mentalities). As the founders of the Annales School, Lucien Febvre, and Marc Bloch were among the first scholars to study the history of mentality. Both of them are familiar to Chinese scholars. Specifically, Febvre's *Martin Luther: A Destiny* explored the mentalities and collective psychology of German society during the 16th century, and Bloch used psycho-historical methods occasionally in his *The Historian's Craft* (Chen, 2003, p. 62–63). Similar to its counterpart in the United States, the research alongside “history of mentalities” in France also made their analysis from the perspective of individual–group or childhood–family. Books such as *L'enfant et la vie familiale sous l'Ancien Régime* (Ariès, 1960/1962) and *The Parisian Sans-Culottes and the French Revolution, 1793-4* (Soboul, 1964) are the typical illustrations of psychohistory in France. But the difference between the French research and the American version is that the former usually analyzed the psyches of a certain figure, historical event, or social group at the micro-level. *The Cheese and the Worms* (Ginzburg, 1976/2013) and *The Great Cat Massacre* (Darnton, 1985) are two representatives of the microhistory in French psychohistory.

Historians of Chinese psychology have merely thought about how psychohistory fits into the broader history of Chinese psychology (Petzold, 1987; Blowers, 2006; Shen, 2006; Hsueh and Guo, 2012; Gao, 2019). When reconstructing the history of Chinese psychology, they do not pay much attention to psychohistory. In view of such reality, the historiography of psychohistory in China should be traced to fill this gap. Undoubtedly, psychohistory in China is influenced by the United States and France, but it is inaccurate to claim that psychohistory in China is simply their follower of them. As we mentioned above, psychohistory here is neither a realm with nearly deterministic power as Isaac Asimov (Thomson, 1996) painted in his science fiction nor a particular field in which Freud's methodology of psychoanalysis is predominant, but involves the combination of psychological and historical scholarship (Elovitz, 2018, p. 8–9). Additionally, in the same vein, psychohistory in China also refers to the use of various psychological theories and methodologies in historical studies. In the following sections, we will take stock of the development of psychohistory in Chinese history with proper periodization, especially in modern times, and then seek to reveal its limitations and possibilities of it.

## The four stages of the development of psychohistory in China

### Stage #1: Before 1902, the pre-scientific stage of psychohistory

As regards the first stage, people should bear in mind that psychology has never been far from historical research in China. The study of history from the perspective of psychological analysis, with its focus on the intrinsic motivations of both individual and group behavior, began as early as the Western Han Dynasty from the historian Sima Qian (Song, 2008, p. 94). In his *Shih Chi* (Records of the Grand Historian), Sima Qian revealed the various psychological activities of different groups, such as emperors, nobles, generals, merchants, Confucian scholars, rangers, and advisors for later generations. This can be regarded as a potential form of psychohistory (Mao, 1992, p. 34). In ancient Chinese history books, psychological factors were frequently employed in explaining the rise and decline of a dynasty. The personalities of rulers and the ruling class were usually considered one of the most important factors that determined the outlook of the empire. When the country was prospering, historians usually attributed it to the virtue of the emperor. In contrast, when the country was suffering from natural disasters, invasions, or riots, the moral degeneration of the ruler or the government officials was always responsible. For example, Emperor Taizong (598–649) of Tang, who is generally regarded as the synonym of “great emperor” in Chinese history, created the “Reign of Zhenguan.” For his success, historians attributed mostly to his good character and personality. He was described as discerning, broad-minded, and diligent (Wu, 1987), and therefore, led China to a golden age. In contrast, those infamous emperors were blamed by historians for their immorality. Emperor Gaozong (1107–1187) of Song was accused by historians of his cowardice and greedy personality which caused the military failure of China. Mao Haijian, a Chinese historian, pointed out that the “mode of the treacherous” was the main narrative mode in traditional Chinese history (Mao, 1995). The development of history was interpreted as the conflict between “the good guys” and “the bad guys,” which decided the destiny of the country. Personalities of historical figures were the last resort in explaining the ups and downs of the country. However, in spite of the emphasis on the personalities and characters of historical figures, it still can hardly accord with the view of “psychohistory” in the modern sense. That is why this stage of psychohistory was described as “pre-scientific”.

### Stage #2: 1902–1949, the introduction of psychohistory

The second stage marked the introduction of psychohistory in its modern sense. The major contribution of this stage was the creation of psychohistory theory in China. With a series of military failures and unequal treaties since 1842, some of the Chinese intellectuals realized that they should follow Western science, technology, and even arts and humanities, including psychohistory, in order to save China from backwardness. Chinese historians tried to innovate the methodology and theory of history for the sake of the modernization of China. At the beginning of the 20th century, through the writings of Japanese scholars, Chinese academics had a preliminary understanding that psychology could be helpful for historical research (Zhang, 2002, p. 51). As early as 1903, Haosheng Li put forward this point of view in his translation of *Introduction to History* (Shigaku genron) by Ukita Kazutami, a professor at Waseda University in Japan (Zou, 1999, p. 27). Qichao Liang, who was one of the most eminent scholars in the late Qing Dynasty, was considered the pioneer of psychohistory in China as he admitted the necessity of combining history with other disciplines, including psychology. In 1902, he published a programmatic article named *The New History*. In this article, Liang tried to re-define the nature, scope, and function of Chinese history as well as the relationship between history and other disciplines. Liang (1902/2014, p. 96) stated that “the theories of the sub-disciplines under philosophy such as ethics, psychology, logic, rhetoric......are also usually related to history indirectly.” Psychology was listed as one of the newly introduced disciplines that could facilitate the formation of modernized history. In his *Research Methods in Chinese History*, Liang (1921/1995) stressed more about the importance of psychology.

“Strictly speaking, if people want to explain history with sole cause-effect mechanism, it will incur unpredictable drawbacks. Why? History is created by the power of the mind which changes freely and unprescriptively. The power of the mind as well as history it creates is not completely dominated by physical or mathematical cause-and-effect law.” (Liang, 1921/1995, p. 151).

It showed that the thought of Liang eventually focused on the influence of the power of the mind and the role of psychological processes in history. In this regard, it can be said that Qichao Liang was one of the earliest scholars in China who advocated the study of historical changes by analyzing social psychology (Lu, 2008, p. 11). To be more specific, Liang (1921/1995) tried to analyze the Boxer Uprising from a psychological perspective. He believed that the Boxer Uprising was caused by two kinds of mentalities, (1) Anti-foreign sentiment, which derived from self-defense and egoism, and (2) Superstition, which is caused by the lack of science and the manipulation of politicians. These two mentalities were provoked by the stimulation of foreign invasions and the corrupted governance of the Qing. As the Qing government was unable to resist the invasion of Germany, Russia, and Japan, the doctrine of “self-strengthening” prevailed among people. However, with the failure of political reform, the relations between Qing and other Powers worsened. People in several provinces organized themselves into war with these Powers in the form of a secret society. The conservative officials in the government connived with the spread of anti-foreign sentiment, which finally led to the outbreak of the Boxer Uprising. It was an outstanding trial of using psychology in history research. Liang's appropriation of psychological analysis also marked the emergence of the vague concept of psychohistory on the soil of China.

The early version of psychohistory pioneered by Liang was soon furthered by other scholars who had been exposed to Western social sciences while studying abroad, including Qianzhi Zhu, Ping-sung Ho, and Qiuyuan Hu, to name a few. Qianzhi Zhu, a famous historian and philosopher, was also notable when recounting the development of psychohistory in China. Zhu, inspired by G. Hegel and A. Comte, invented his historical theory on the basis of rationalism and psychologism, for which the latter is actually more valuable. “It is because historical materialism is the combination of Hegelian dialectic and materialism, only the pathogeny during social evolution can be seen. In contrast, the law of social evolution can be discovered *via* psychologism” (Zhu, 1933/2002, p. 137). It is difficult to conclude that his understanding of historical materialism is accurate, but it indeed reflected the fact that Zhu realized the potential of applying psychological methods to history. He argues that in addition to studying various situations of social life—such as family, population, urban, and economic problems—historical research must also pay attention to psychological methods (For Zhu, this includes psychopathology, mass psychology, and Wundtian psychology) to find out historical procedures from the phenomena of human psychology (Wang, 2015, p. 131).

In 1922, Ping-sung Ho, a notable Chinese educator, writer, and historian, chose *The New History* (Original work published in 1912) authored by James. H. Robinson as the textbook in his history courses at Peking University. Within a year, he translated it into Chinese, which became the first book concerning psychohistory by a Western scholar known to a Chinese audience and was hailed as “the first work on the theory and methodology of Western historiography translated by Chinese historians, which is of great importance” (Huang, 1981, p. 16). In *The New History*, Robinson (1912/1922) stressed that a full understanding of history could only be possible when it is combined with other disciplines. As he stated in the book:

The “New History” is escaping from the limitations formerly imposed upon the study of the past. It will come in time consciously to meet our daily needs; it will avail itself of all those discoveries that are being made about mankind by anthropologists, economists, psychologists, and sociologists —discoveries which during the past 50 years have served to revolutionize our ideas of the origin, progress, and prospects of our race (Robinson, 1912/1922, p. 22–23).

Instead of confining itself to a narrow sense, historical research should engage in interdisciplinary research. This idea was echoed among Chinese historians such as Dazhao Li, a Marxist theorist and the early leader of the CPC. He advocated the necessity of combining history with other disciplines. In his *Essentials of Historiography*, he proposed that:

Historical theorists who look for the formation of theories must carefully study the “particular facts” first. Only these examined results can be used as the foundation of historical theories. At the same time, they should take into account the findings of biology, archeology, psychology, sociology, and other humanities so as to examine the results of written, oral, and theoretical history (Li, 1924/2011, p. 83).

However, it should be noted that as a Marxist, Li prioritized economic activities in historical research. “This is the explanation of historical materialism. Interpretation of this kind does not trace to the effects of ideas but material basis because the change of ideas is dominated by the material circumstances.” (Li, 1924/2011, p. 23) It indicates that Li tended to seek interpretation of history *via* the investigation of economic activities while psychological activities were regarded to be their outcome.

Another important figure, Qiuyuan Hu, despite his controversial political identity, was recognized as the first generation of historians who applied materialism to research. In his, *The Outline of Historical Philosophy*, which emphasized the significance of different social sciences for historical research, Hu (1940/1948, p. 63) commented that “ranging from human psychology to zeitgeist, their influences on history and culture formation are undeniable.” Although he also pointed out that psychology “is not sufficient to explain the progress of social history” (Hu, 1940/1948, p. 63), it at least showed that psychology had caught the attention of materialist theorists in the field of historical research.

In the 1930's, several translations related to psychoanalysis also appeared in China. Guangqian Zhu is considered the first scholar to introduce Freud's theories of psychoanalysis to Chinese readers through his book *Abnormal Psychology* (Zhang, 2002). Subsequently, Juefu Gao translated Freud's book *Introductory Lectures on Psycho-Analysis* (published in German 1916–1917, in English 1920) and presented it to Chinese readers in 1930. Si Cai translated Freud's lectures in the United States and published them in journals to introduce to the Chinese audience Freud's thought in the 1930's (quoted in Wang and Bai, 2013). If psychobiography is viewed as an individual orientation of psychohistory (Xiao, 2010), the introduction of psychoanalytic methodologies laid the foundation for the advancement of psychohistory and psychobiography in China.

Despite the above-mentioned efforts to advance psycho-historical research in China, it should be reminded that in the theoretical experiments of these scholars, although they generally believed that psychology was one of the ingredients that could help create the “new” Chinese historical research, their vision is a comprehensive and modernized Chinese historical writing, rather than the exact discipline of psychohistory itself. However, their emphasis on the interdisciplinary nature of historical research made possible the further development of psychohistory.

### Stage #3: 1949–1978, the tortuous and lopsided development of psychohistory

In the third stage, the development of psychohistory underwent a series of progress as well as setbacks. Since 1949, Chinese psychologists and historians shifted their research to the Soviet paradigm and tried to participate in the socialist transformation at that time (Gao, 2020). The Soviet paradigm of psychology, such as its version of Pavlovian behaviorism, was adopted and emulated by Chinese psychologists. Historical materialism was also elevated to be the widely accepted principle of academic research. The ideology of Marxism–Leninism achieved major dominance in different disciplines. The Central Committee of the CPC (1958) promulgated the *Instruction Concerning Educational Work*, which stipulated that “Education must serve proletarian politics and be combined with productive labor.” Therefore, apart from theoretical research, psychologists were also looking for opportunities to put their theories into practice. Research in the field of psychology had made some progress despite the interruption of the “Criticizing the Bourgeois-oriented Psychology” movement of 1958. Social psychology provided psychohistory with new dynamics *via* its exploration of the class character (jie-ji-xing). Class character became a central topic in social psychological research in the 1950's. Richang Cao, who obtained his Ph.D. in Psychology from the University of Cambridge, stressed the importance of class character as the theme of psychological research. He stated that:

In a class society, people of the same class share the same characteristics, which makes them different from people of other classes. This is class character. Psychology should also study personality and class character. Personality is related to physical condition but is mainly formed in life experience. Personality is the reflection of social and life relations. Class character is the reflection of social status (Cao, 1959, p. 247).

Class character became an important factor in mediating the relationship between history and psychology at this historical stage. In the study of the evolution of class character, it was believed that psychologists and historians could discover the law and features of the psychological activities of human beings, hence manipulating them to serve the needs of the country. However, the development of psychology and history was eventually affected by the more and more pan-politicized atmosphere since the late 1950's. According to the prevailing view of this period, Marxism–Leninism emphasized the decisive impact of socio-economic relations on which political and cultural spheres are founded while the psychological activities of human beings are classified to be the superstructure. The Marxist historiography system, in many cases, ignores the psychological level in the historical process, thus creating a vacuum of historical materialism, which emphasizes economic factors over psychological factors, the role of groups over the role of individuals, and the rational components over the irrational components (Wang, 1989, p. 25). Therefore, psychology was regarded as a less important, less fundamental, and idealistic discipline. In this vein, the class analysis method rose to be an effective fundamental methodology. It was encouraged and generally accepted by Chinese psychologists and gradually replaced the traditional methodology of psychology that was considered idealism. The extreme consequence of this practice was that psychological research (including the possible attempts of psychohistory) was halted during the Cultural Revolution. Under such circumstances, psychohistory would not be able to flourish until the next historical stage raised its curtain.

### Stage #4: 1978–present, the revival of psychohistory

Finally, with the beginning of the Reform and Opening-Up Policy and then the introduction of new approaches to history, the psychohistory was revived, which signified its fourth stage. Various schools and methodologies of historical research, such as New Cultural History, École des Annales, cliometrics, microhistory, and psychohistory, entered the scope of analysis among Chinese historians. Scholars during this period had a clear disciplinary consciousness of psychohistory. They proposed that “it is entirely necessary to create a discipline of ‘historical psychology',” thus opening a new chapter in the study of Chinese psychohistory (Cai, 1983). This awareness is reflected in the exploration of possible psycho-historical research in China on the basis of translating and publishing a large number of related foreign works, including works by several influential historians (L. Febvre, O. Pflanze, Thomas Kohut, and Richard Schoenwald), and books of the same type such as *The New History* (deMause, 1975/1989), *Shrinking History: On Freud and the Failure of Psychohistory* (Stannard, 1980), *Montaillou, an Occitan Village from 1294 to 1324* (LeRoy Ladurie, 1975/1979), *The Great Cat Massacre* (Darnton, 1985), and *The Cheese and the Worms* (Ginzburg, 1976/2013).

Compared with the orthodox interpretation of Marxism during the 1960's, the view of psychohistory provides a new perspective to Chinese historians and psychologists. “First, it reverses the relationship between physical and psychological reality, so that instead of material progress setting the pace of history and somehow dragging behind the psyches of its actors, human psychology is made primary, setting Marx on his head and Hegel back on his feet – and material reality is viewed as primarily the outcome of man's decisions, past or present, conscious or unconscious. Second, the major basis for historical change is the interrelations of persons, not forgetting the relations between generations, and man is viewed for the first time not as *homo faber* but as *homo relatens*” (deMause, 1975/1989, p. 8–9).

On the basis of the translation and introduction of Western psychohistory, Chinese scholars also began to apply it to Chinese issues. Ma (1986) analyzed the psychological features of Chinese merchants and stated that they had four characteristics, including (1) a sense of crisis, (2) self-respect, (3) a sense of backwardness, and (4) a sense of belongings, which led to their tendency toward compromise in the revolutionary movement. In his *People's Psyche under the Rule of Autocracy*, Xie (1990) believed that the autocracy in China caused the emperors' capricious characters. The affiliated government officials, therefore, had to become Machiavellian and conservative in order to survive in power struggles. Other remarkable pieces include *Dream of Hundred Years: The Psychological Change of Contemporary Chinese Intellectuals* (1988) by Yan Zhou, *The Metaphysics and Intellectuals' Mentalities during Wei and Jin Dynasties* (1991) by Zongqiang Luo and *Frustrated Travelers: The Mentalities of Chinese Contemporary Intellectuals* (1995) by Zhang.

Throughout that period, the progress of psychobiography in China also apparently started. Since June 2012, Jianhong Zheng et al. have promoted the institutionalization of psychobiography in China (Shu, 2018), which signifies that the tradition of psychobiography has been established in China.

The efforts of Chinese scholars mentioned above are undoubtedly strong evidence to show that the application of psychohistory has already engaged with the traditional approaches to both history and psychology and expanded their research scope. It would not be surprising if Chinese psychohistory can achieve more substantial and in-depth development in the future.

## What is missing in Chinese psychohistory

Despite its recent development, there are also some challenges to Chinese psychohistory. First, Western theories are often not something that can be appropriated directly, and their compatibility with Chinese issues needs to be further examined. The indigenization of Chinese psychohistory still has a long way to go. It is not unusual that people ask whether concepts like “Oedipus Complex,” “Castration Fear” can be applied to Chinese people as psychoanalysis emerged during the Victorian Age. Therefore, it is necessary for researchers to bear in mind that the differences between China and the West in psychological, cultural, economic, and geographical aspects might be incommensurable. The West as *methodology* is a good heuristic reference for solving China's problems, but if the West is used as a *problem* itself, it is possible to go astray in Western-centrism. Some Chinese scholars have already tried to reconsider and even supplement Western theories by introducing Chinese concepts and variables. For example, the concept “Qi,” is a feeling of not being respected and treated justly, and also a sense (even spirit to some extent) of justice and respect for others which may cause corresponding actions actually in one of its several meanings (it thus has various translations in English, such as “breath,” “ether,” “air,” “temperature,” “energy,” and “anger”, but none of these could fully express the idea it refers to in the context of Chinese), is believed to be an important factor that Western theories regarding social mobilization cannot perfectly cover when understanding contentious politics in rural China (Ying, 2011). Yang (1994/2004) stated that the hereditary factors (such as social, historical, and intellectual ones) of a particular country or society, on the one hand, affect the psyches and behaviors of the subjects, and on the other hand, they also affect the issue, methodology, and theory of the researchers. Therefore, only when psychology, as well as psychohistory, is built upon these mutual factors can the theory serve the local people. Whether introducing Western theories into China or extending Chinese theories to the world, the issue of indigenization is a priority for both sides. Whatever the theory, it needs to be adapted to the local context of the nation and the region.

Second, it is necessary for psychohistory in China to be more internationalized and contribute its own ideas to the solution of some universal problems. Admittedly speaking, the uniqueness of Chinese culture and psychology requires the development of a corresponding Chinese psychohistory. Western formal rationality emphasizes the calculability of means and procedures that can be used to pursue personal goals and pays attention only to value-neutral facts (Hwang, 2012, p. 30). Fundamentally different from formal rationality of this kind, Confucianism contains a unique concept of “mind and disposition” (xin-xing) which requires the realization of various universal ethical principles in the life world as concrete experiences through personal practice, with humaneness (Li, 2010), immanent transcendence (Stock, 2021), and ideal personality (Ge, 2020) as the ultimate value of being. But it cannot satisfy itself with this and stop there. Chinese indigenous psychohistory will first be able to inspire other East Asian countries as they are all within the Confucian cultural circle. In the long run, a truly developed Chinese psychohistory has the potential to make greater contributions to a “universal” or “world” psychohistory. For example, Zehou Li put forward his theory of “culture-psychological structure,” a term he coined to distinguish itself from the Western cognitive model of “psycho-cultural structure.” “They (the Westerners) explain culture in terms of psychology, while I explain psychology in terms of culture, and think that culture builds up unconsciously into psychology. Therefore, the cultural structure is closely related to the psychological structure (specifically, such as mode of thought and behavior, emotional state, and aesthetic taste)” (Li, 1999, p. 475).

Moreover, apart from psychoanalysis, psychohistory in China needs to absorb in-depth other emerging schools of psychology, such as humanistic psychology and behaviorism. In fact, with the rise of consumerism in China after the reform and opening up, behaviorism can be used to analyze the daily behavior of Chinese people. In the meantime, humanistic psychology has already been applied to analyze the pursuit of self-realization of Bang Liu (Shu, 2015), the founding Emperor of the Han Dynasty, and Sima Qian (Dang and Duan, 1993). There is even an attempt to use Piaget's genetic epistemology to analyze the dynamic development of Mao Zedong's cognitive structure (Xiao, 2005).

Finally, psychohistory in China also needs to strive for further institutionalization in order to establish its own disciplinary status and expand its influence. For now, it is often considered only as an interdisciplinary research method combining history and psychology, a new historiographical method in accordance with modern Western historical research to analyze various historical phenomena (Zhang, 2010, p. 108). Few universities in China provide courses in psychohistory for undergraduates. Researchers usually come from different departments and conduct research according to their personal interests. Thus, it is inevitably unfavorable to the teaching and learning of psychohistory (Liang, 2013). Therefore, a higher degree of institutionalization, including professional associations, seminars, journals, and research institutes, is indispensable for the further development of psychohistory in China. Nevertheless, it does not mean that psychohistory should become a self-isolated kingdom of inquiry; on the contrary, it is still necessary to (1) be open to other realms of knowledge like sociology as Smelser (1998) did, and ethics as Erich Fromm (1941/1969) did, to name but a few, and (2) consider the macro-massive social changes, especially economic changes not as mere background, but as actual variables that affect and are affected by psychological consequences (Fraad and Fraad-Wolff, 2014).

## The practical relevance of psychohistory to China today

As an emerging discipline in China, psychohistory has a huge potential role in both historical research and social issues. First, psychohistory overturns the traditional notion of historical figures as rational people, which was commonly found in Chinese historical studies. In classic Chinese historical studies, historical figures are often regarded as *Homo oeconomicus*, and their decision-making process is considered to be based on rational judgment. However, psychoanalysis revealed that human beings are “neurotic animals” to a certain extent since people often do not behave as reasonably and calmly as we think but instead show a propensity toward negativity, anxiety, and self-doubt. Psychohistory can, therefore, help explain some seemingly irrational phenomena in Chinese history. For example, by tracing the life experience of Emperor Qin Shihuang, Wang (1999) pointed out that his suspicious and over-sensitive character was the result of his miserable and unrestful adolescence. The abnormal personality development of Qin Shihuang led to his egoism and tyranny during his regime. As a result, an empire that was going to produce emperors of all ages perished in two generations. Similarly, Zhang (2014) pointed out that Emperor Guangxu had Recurrent Brief Depression (RBD) which led to his improper decision in the Sino-Japanese War and 100 Days of Reform. In addition, Yan Yuan, an important thinker in the early Qing Dynasty who expected himself to be a sage, was deeply suppressed by his grandfather and was in a state of psychological tension for a long time, and in terms of scholarship, he unconsciously questioned the repressive doctrine of Chu Hsi. However, he later learned that his grandfather was actually an adoptive grandfather, and meanwhile, he turned intellectually from Cheng-Zhu Neo-Confucianism to Confucius and Mencius' classical one. From a psycho-historical point of view, the simultaneous occurrence of these two processes is not accidental. Knowing the truth of his ancestry enabled him to solve his identity crisis by recognizing and returning to his origin at the two levels of bloodline and academic genealogy at the same time (Wang, 2018).

Second, for some historical events in China, psychohistory can provide key insights into explaining their origins and mechanisms. The topic such as the activities of secret societies and local religions in the early Qing Dynasty, the Taiping and Boxer uprisings in the late Qing Dynasty, and the Cultural Revolution in the 1960–70's are ones for which psychohistory can provide new explanations. For example, more and more historians are turning to psychology to find new explanations for the outbreak of the Cultural Revolution from the bottom of society, including the various characteristics of the metamorphosis of the masses such as fanatical worship, morbid fear, grandiose delusions, negative-conservative attitudes, naive blind obedience, and distorted rebellious mentality. Furthermore, there are scholars who (Wang, 2012) inspected the social psychological characteristics of the Cultural Revolution period from four perspectives: (1) the psychological status of ordinary individuals, (2) the social motivation, (3) the psychological quality of the modern Chinese nation, and (4) the special social environment. In addition, profit-seeking and herd mentality in the process of personal socialization and the fixed mindset among the group of leaders can also be found, which were employed to analyze the psychological origins of the Great Leap Forward (Rao and Pang, 2003, p. 33–35).

Third, psycho-historical methods can also be applied to analyze and document essential historical changes and social events that occur in contemporary China. Notwithstanding, either sociology or psychology has incorporated an analysis of these major social problems. Hence, psychohistory should preserve sufficient historical materials for the future and provide corresponding psychological analysis. For instance, with the advent of the two-child policy, family planning has gradually retreated from that stage of history. Yet, the issues brought about by the family planning policy are affecting every aspect of Chinese society. Those issues include the pension of the only child (Sui et al., 2022), the imbalance of the sex ratio (Chen et al., 2007), and mental health problems of the only child (Feng, 2002), among others. The impact of family planning involves numerous objects, including individuals, families, society, and culture. For individuals, the impact of family planning involves distinctive stages from childhood and adulthood to old age. Consequently, in accordance with divergent research objects and distinct sub-problems, psycho-historical methods can be adopted to study the corresponding targets. One recently proposed theory called Psychodialectical Cultural Reason Theory (Bilge, 2022) can be applied to analyze social and cultural phenomena. This theory directly combines psychodynamics and social processes, enabling a structural analysis of collective consciousness. Accordingly, this theory may be utilized to inspect the concept of “preferring sons to daughters” under the family planning policy. Besides, Adler's individual psychology and Erikson's self-identity theory can likewise be utilized to comprehend the mental health issues of only children. This is a crucial subject that is faced by psychologists, historians, and sociologists.

Finally, psychohistory has important practical significance for understanding and dealing with not only the current psychological problems of Chinese people but also the wider range of issues concerning social organization, such as the transformation of bureaucracy and hierarchy (Allcorn and Stein, 2020). China's dramatic modernization process since the 19th century has raised a series of psychological and sociological issues that psychohistory can at least help understand, especially the formation of modern Chinese mentality and values, as well as the emerging corporate culture like “996” (the schedule of working from 9 a.m. to 9 p.m. for 6 days per week) and its consequences. Yang (2008) pointed out that the Chinese people have the following traditional characteristics: diligence, humility, familism, and hierarchy. However, after the rise of the market economy since the late 1970's, these traditional characters have encountered serious challenges, and there have been some psychological problems and abnormal behaviors that cannot be ignored in Chinese society, including anxiety, depression, corruption, and fraud. The conflict between economic and even political liberalism, consumerism, and traditional Chinese morality has led to a crisis of belief for many people, and changes in social policies have also induced certain social groups who find themselves disadvantaged, resulting in a sense of relative deprivation and various pathological behaviors. For example, the historical changes in the mentality of the working class in northeast China are a case worth studying. The restructuring of many state-owned factories in this old industrial area has produced a large number of laid-off workers. Many of them have suffered from social and psychological difficulties, such as a sense of injustice, instability, and alienation. These psychosocial dilemmas are often manifested by anxiety, depression, irritability, restlessness, and even misanthropy or passive and violent confrontation (Huang, 2009, p. 45–48). In this case, psychohistory helps to provide a historical analysis of changes in workers' mentality because of its theoretical perspective on group psycho-historical changes. Psychohistory will further contribute to the development of policies to reduce related behavioral aberrations. Taken together, it demonstrates the value of psychohistory in the policymaking process and, ultimately, in dealing with current social issues in China.

## What can the Chinese psychohistory bring to the world?

To answer this question, one needs to be focused on the distinctions between China and the West. According to some scholars, the family is one of the four objects of psychohistory (Chen, 2003; Yang and Shu, 2018). First and foremost, since ancient times, the family has been the concentration of Chinese traditional culture. For example, as an old Chinese proverb indicates:

After his desire for material comfort was clear away, then his innate knowledge would be established; once his innate knowledge is established, then his thought would be sincere, once his thoughts are sincere, then his mind would be upright; once his mind is upright, then he himself would be cultivated; once he himself is cultivated, then his family would be regulated; once his family is regulated, then his state would be administered well and all the lands under heaven would be peaceful and tranquil (Dai, 2017, p. 326, with minor modifications in translation).

This suggests that the family is the basic element of Chinese society. Additionally, it is a microcosm of both society and nation. Zhang (2017, p. 49–50) claims, compared with Heidegger's concept of the family, in Confucianism, the concept of the family appears more vivid and affectionate; thus, it has ultimate relevance. Its ultimate significance comes from human relations, namely “Ren Lun” celebrated as “Qin Qin” in Chinese (Zhu, 2019), which signifies that (1) one needs to regulate the relationship of his sons and grandsons near and distant to show the law of his affection to his relatives, (2) love his kinsmen, and (3) ties of blood (Dai, 2017, p. 173–174). In recent years, “family disorganization” has emerged in the West countries (Deng, 2008. p. 279–281). This consequence has led to social problems such as youth gang membership (Sharkey et al., 2011), volatile substance abuse (Broi et al., 2015), antisocial behavior (Ford, 2009. p. 251–279), and juvenile delinquency (Gerard et al., 2014). Additionally, “Qin Qin,” as a representative of the traditional Chinese cultural concept of family relations, assists in creating a good parent–child relationship. In turn, it reduces the occurrence of the above problems at the family level. Hence, western scholars should have a fundamental understanding of Chinese culture, especially Confucianism when they study the attachment to a Chinese family. Besides, in addition to understanding the family's special relationships using the perspectives of psychoanalysis such as the Oedipus Complex and the Erlektra Complex, western scholars can also examine the family's closeness by employing traditional Chinese concepts such as “Qin Qin.”

Second, China has developed distinctive sociological theories to explain human relationships. Fei (1948/2019) put forward the famous concept of “Cha Xu Ge Ju” (the differential mode of association). It is pointed out that the traditional social network of the Chinese is tied by blood, kinship, and geography. People in social relations are like ripples created by throwing a stone into the water. This theory describes the characteristics of traditional social structure and human relationships in China. But in the Western world, the crisis of “atomization,” and consequently social atomization, arose from the very beginning of modern society (Tian and Lyu, 2010). Individuals are abstractly linked by market exchange, sometimes unable to form organically connected communities. On the contrary the Chinese disparity pattern provides a framework for the development of embodied, authentic human relationships. Although compared with the traditional situation, China's social structure has undergone tremendous changes, the theory still has strong explanatory power (Bu, 2003). Accordingly, when psycho-historians need to study human feelings and realities of the Chinese, theories of this kind are of indigenous compatibility.

Third, not only does China have a long history but also it has a long tradition of recording history. Since ancient times, the Chinese have possessed a widespread appreciation for history, especially for the significance of long-term history. Clearly, history plays a significant role in both Chinese political life and daily life. For example, in terms of political life, China produced the so-called official *Twenty-Five Histories*, a systematic, comprehensive, and authoritative record of civilization covering a period from 3000 BC to the Qing dynasty which ended in 1911. In terms of daily life, because the Chinese attach great importance to what posthumous evaluation they would receive from others, they are not only cautious in their words and deeds but also leave information about themselves deliberately through diaries, biographies, epitaphs, genealogies, and so on. As an old Chinese idiom says, “Gai Guan Lun Ding” (final judgment can be passed on a person only when the lid is laid on his coffin), on the condition that you consider issues on the basis of their influence over a long period and even on the judgment of others after your own death, the way you think and behave will probably change accordingly. The emphasis on the history of Chinese, on the one hand, provides rich materials for the study of Chinese psychohistory, and on the other hand, inspires us to take into account the long-range diachronic variables when studying Chinese psychology and behavior. In comparison to the logic of daily activities in the West, this is also a distinctive aspect of Chinese psychology.

## Conclusion

In general, psychohistory in China has undergone four historical stages of development. Since the late Qing Dynasty, Chinese historians have been seeking to modernize the theories and methods of historical research, and psychology, as an important component of modern disciplines, attracted their attention and was absorbed in their visualization of a modernized disciplinary system. Despite the interruption of political movements during the 1950's and the Cultural Revolution, psychohistory has made developments both theoretically and practically by and large. In the meantime, there are still several limitations to which psychohistory in China is confined.

However, psychohistory, as a potential discipline, has remarkable significance in analyzing the psychological evolution of the Chinese people. In the foreseeable future, the horizon of Chinese psycho-historical research will be further broadened. However, psychohistory, as a potential discipline, has remarkable significance in analyzing the psychological evolution of the Chinese people. In the foreseeable future, the horizon of Chinese psycho-historical research will be further broadened. Chinese psychologists and historians are to explore the issues of traditional Chinese psycho-historical thinking from different perspectives such as philosophy, science, culture, and anthropology. On these bases, they will be able to realize the creative redevelopment of psycho-historical research in the dialog between ancient and modern, as well as between China and the West. At that time, Chinese psycho-historical research will no longer be psychohistory simply *in* China but will become psychohistory *of* China.

## Data availability statement

The original contributions presented in the study are included in the article/supplementary material, further inquiries can be directed to the corresponding author.

## Author contributions

All authors listed have made a substantial, direct, and intellectual contribution to the work and approved it for publication.

## Funding

This work was funded by Major Project of National Social Science Foundation of China [21ZDA072].

## Conflict of interest

The authors declare that the research was conducted in the absence of any commercial or financial relationships that could be construed as a potential conflict of interest.

## Publisher's note

All claims expressed in this article are solely those of the authors and do not necessarily represent those of their affiliated organizations, or those of the publisher, the editors and the reviewers. Any product that may be evaluated in this article, or claim that may be made by its manufacturer, is not guaranteed or endorsed by the publisher.
